# Editorial: Exploring shared intentionality: underlying mechanisms, evolutionary roots, developmental trajectories, and cultural influences

**DOI:** 10.3389/fpsyg.2023.1332592

**Published:** 2023-11-28

**Authors:** Tomas Persson, Gabriela-Alina Sauciuc, Valentina Fantasia, Kim Bard

**Affiliations:** ^1^Department of Philosophy/Cognitive Science, Lund University, Lund, Sweden; ^2^Centre for Comparative and Evolutionary Psychology, University of Portsmouth, Portsmouth, United Kingdom

**Keywords:** shared intentionality, joint action, human development, evolutionary origins, animal cognition, cross-cultural variability, neurobiological mechanisms, socio-cognitive mechanisms

Shared intentionality (henceforth SI) is a theoretical construct that refers to a suite of abilities that enable coordinated, collaborative interactions, and claims that the mechanism to obtain these skills reside in the sharing of mental states, such as attention and goals (e.g., Tomasello, 2019). According to this standard view, SI is a recent human adaptation with no counterparts or precursors among other great apes or any other animals. During the last couple of decades, SI has become an influential concept in research on human social cognition and its development, in theories of human evolution, and even in assessments and intervention programs for children, e.g., with autism. There is, however, controversy surrounding the nature and scope of the SI construct, its ingredient processes and behavioral markers (see e.g., the recent anthological volumes edited by Durt et al., 2017 or Fiebich, 2020). Notions differ regarding what exactly is shared, which behavioral and/or socio-cognitive traits are crucially involved, and how SI can be studied.

The goal of this Research Topic has been to highlight empirical findings and theories that are important for elucidating the evolutionary and developmental foundations of abilities and processes associated with SI, as well as their cross-cultural distribution and variability. The articles included in this Research Topic contributed to achieving this goal with either empirical (human children: Macheta et al., Kasuya and Nonaka; chimpanzees: Hopkins et al.) or theoretical studies (Hawkes, Papadopoulos, Sauciuc and Persson, Skau, Vincini). We have specifically welcomed research on the underlying psychobiological and neurocognitive mechanisms implicated in SI, as well as research on the processes and contexts which support, mediate or constrain SI. In response to this, Sauciuc and Persson review comparative and developmental data on a broad range of putative SI mechanisms postulated to be species-unique, whereas other topic contributions have a more specific focus. Hopkins et al. address genetic, neuroanatomical and experiential mechanisms of joint attention—a foundational component of SI—in a large sample of chimpanzees. They find that gray matter volume in brain areas hypothesized to implement SI in humans, singly account for the chimpanzees' performance in a popular test of joint attention. Kasuya and Nonaka reveal that Japanese toddler pointing is longitudinally associated with attention to the caregivers' face, as well as with caregivers' attention to the ongoing interaction. Addressing the relationship between epistemic- and emotional state comprehension, Macheta et al. find that the ability to understand that different individuals may access objects with different—albeit equally “true”—conceptualizations, predicts emotion comprehension in middle-class Polish children. Skau proposes a theoretical distinction between cognitive mechanisms that generate cooperative group activities and mechanisms that sustain them, as well as making a distinction between joint attention and joint attentiveness. Vincini suggests abolishing the requirements for mental state representation and sharing, suggesting that joint actions are enabled by forming associations between own-others' experiences and bodies. Hawkes advocates a life-history perspective in order to account for the precocious engagements of human infants in cooperative exchanges with others, which is attributed to mechanisms triggered by human-unique life-history features.

The open call for contributions has reached researchers that study SI in the standard tradition as well as those that directly or indirectly contrast it to alternative views. Interestingly, the views critical of the standard view, on both conceptual and methodological grounds, dominated the submissions. Several articles argued against the traditional postulate that SI and cooperation require cognitive abilities specialized for perspective taking and mental state sharing, or against claims of SI human-uniqueness (Hopkins et al., Papadopoulos, Sauciuc and Persson, Vincini). The empirical intractability of the SI-associated constructs was a recurrent motif. Another theme is the criticism of Western-biased empirical and conceptual foundations in the standard view (Hopkins et al., Macheta et al., Papadopoulos, Sauciuc and Persson), which may render the standard view inadequate or, indeed, inapplicable across human populations of the world. Several papers make the point that a way to counter such biases, and thus ensure more representative conceptualizations, is to account for variability at multiple levels, from genetic make-up and neuroanatomy (Hopkins et al.) to socio-economic, ecological and cultural background (Macheta et al., Sauciuc and Persson), and to long- or short-term experiential and interactional effects (Hopkins et al., Kasuya and Nonaka, Sauciuc and Persson). Such data are also crucial in delineating alternative dynamic and/or situated perspectives on joint engagements and cooperation, which are most saliently represented in the papers of Kasuya and Nonaka, Papadopoulos, and Vincini.

Looking at the contributions as a whole it is clear that they highlight a wide spectrum of questions that one can ask about an established influential research tradition such as SI theory, which has largely been defined within the context of tests with human children and non-human great apes, using methodology from experimental psychology. Being critical, as most of the contributions are with respect to the standard view of SI, is especially important when theories are impactful outside academic discussions. SI theory affects how we view social cognition in human infants, in certain clinical groups, in non-human animals, and how we view the evolution of the human species. At a minimum, before putting theory into practice, one needs to ascertain that the research findings are widely applicable to the species or groups that the theories target. This requires a diversification of paradigms beyond those used in a single field, such as experimental psychology. It is therefore necessary to continue to bring together different bodies of research with the aim to promote multidisciplinary exchange, and interdisciplinary research on SI-relevant abilities and processes. Only then can new, more integrative, research ideas and findings be generated and SI theory potentially be revised.

## Author contributions

TP: Funding acquisition, Writing—original draft, Writing – review & editing, Conceptualization. G-AS: Conceptualization, Funding acquisition, Writing—original draft, Writing—review & editing. VF: Conceptualization, Writing—review & editing, Funding acquisition. KB: Conceptualization, Writing—review & editing.
